# Supplementary material for: Methamphetamine modulates functional connectivity signatures of sustained attention and arousal
Source: bioRxiv. 2025 May 21:2025.05.20.655181. Preprint. [Version 1] doi: 10.1101/2025.05.20.655181 (PMC12139791; doi:10.1101/2025.05.20.655181)
Supplement: 1 [file NIHPP2025.05.20.655181v1-supplement-1.pdf]

bioRxiv preprint doi: https://doi.org/10.1101/2025.05.20.655181; this version posted May 21, 2025. The copyright holder for this preprint (which was not certified by peer review) is the author/funder, who has granted bioRxiv a license to display the preprint in perpetuity. It is made available under aCC-BY 4.0 International license.

Supplementary Data

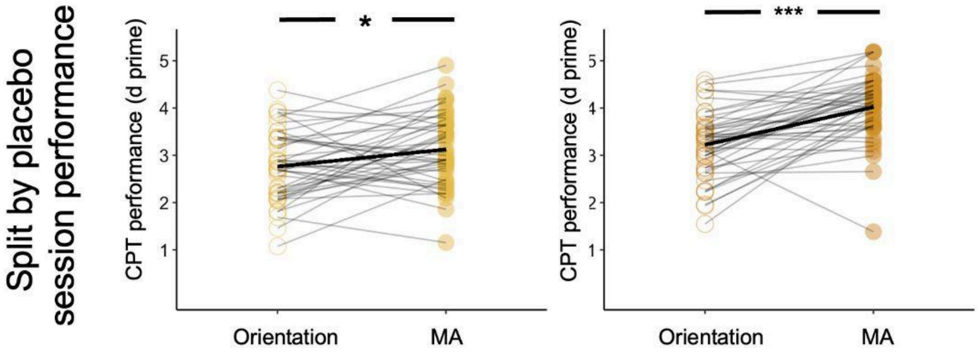

Figure S1. Participants were split into high and low performer groups using a median split based on their CPT performance under the placebo session. Both low performers and high performers have improved CPT performance in the MA condition compared to the orientation session.

bioRxiv preprint doi: https://doi.org/10.1101/2025.05.20.655181; this version posted May 21, 2025. The copyright holder for this preprint (which was not certified by peer review) is the author/funder, who has granted bioRxiv a license to display the preprint in perpetuity. It is made available under aCC-BY 4.0 International license.

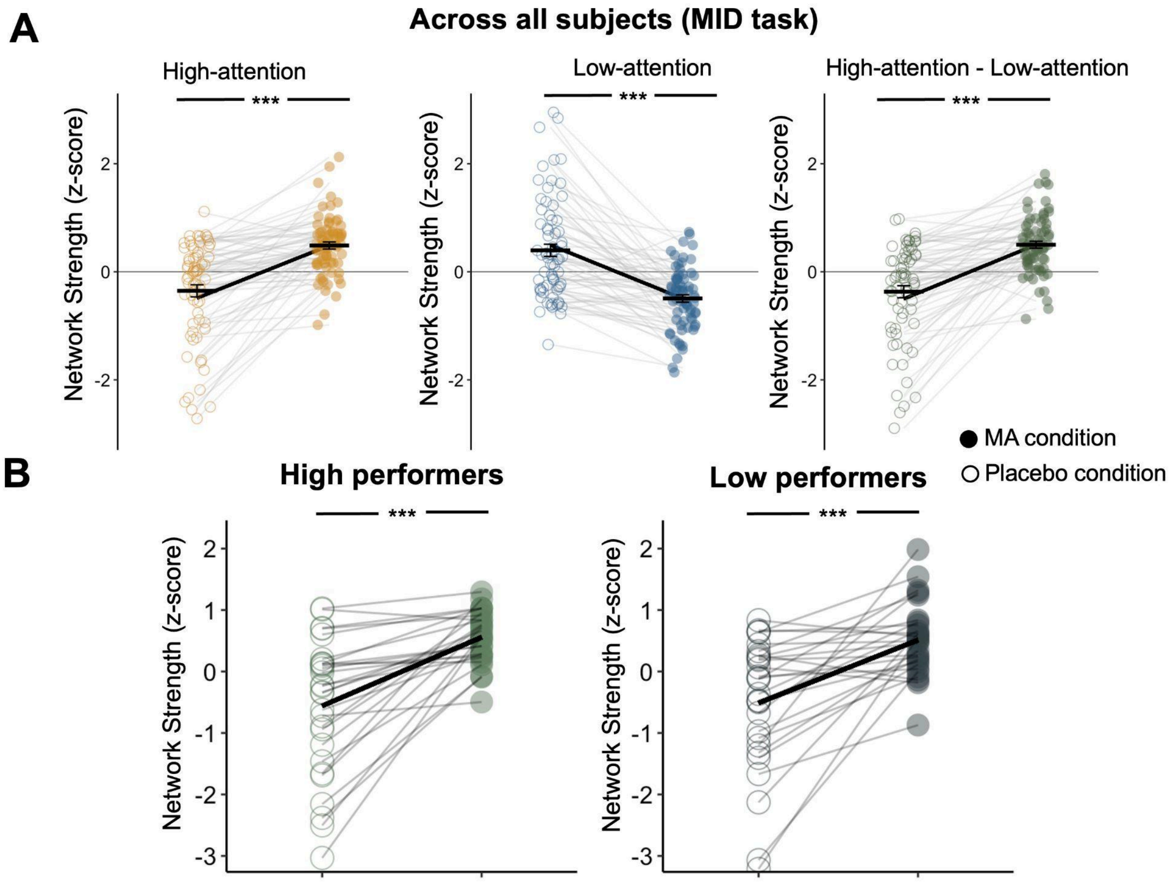

Figure S2. MA administration increased strength in the high-attention network and decreased strength in the low-attention network relative to control conditions. Network strength was calculated during the MID task, and was normalized within each graph for visualization. **B.** Effects of MA on network strength were similar regardless of orientation session performance on the gradCPT. Dots represent individual participants, horizontal lines denote condition means, and error bars represent standard error of the mean.

bioRxiv preprint doi: https://doi.org/10.1101/2025.05.20.655181; this version posted May 21, 2025. The copyright holder for this preprint (which was not certified by peer review) is the author/funder, who has granted bioRxiv a license to display the preprint in perpetuity. It is made available under aCC-BY 4.0 International license.

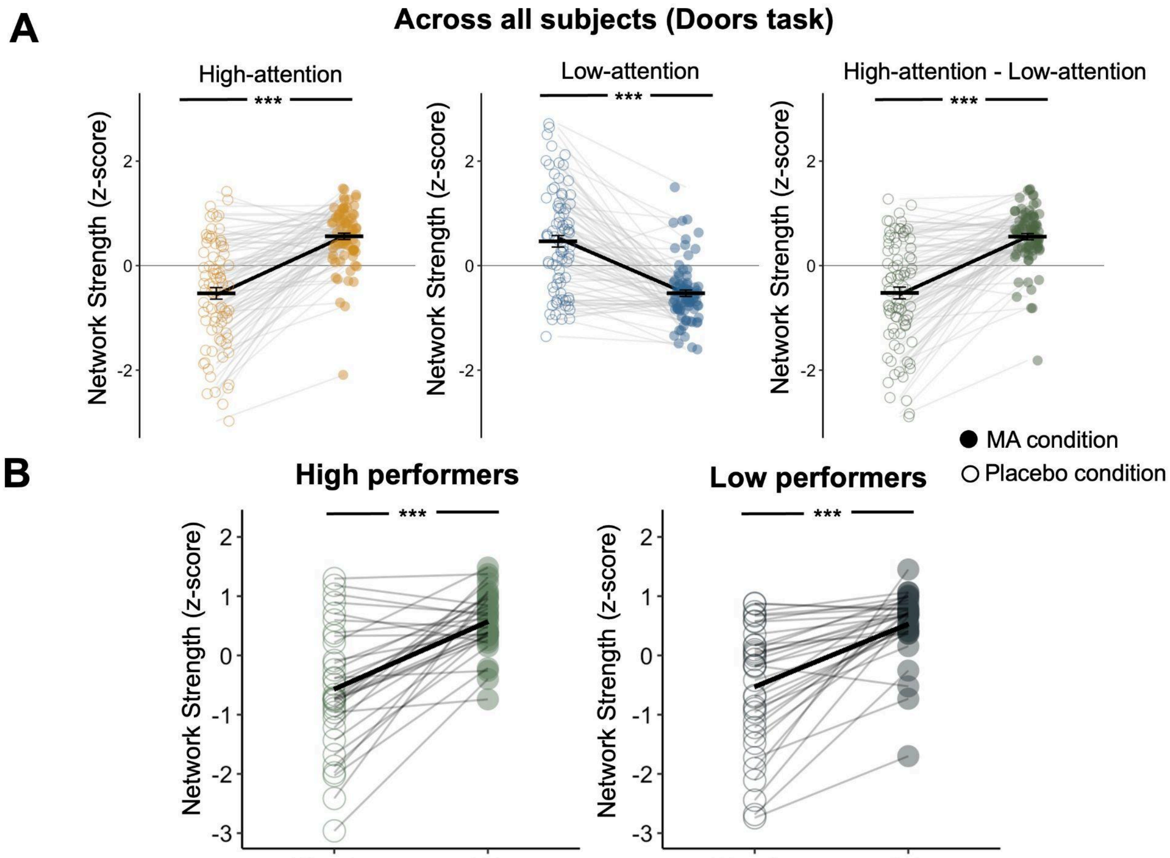

Figure S3. MA administration increased strength in the high-attention network and decreased strength in the low-attention network relative to control conditions. Network strength was calculated during the Doors task, and was normalized within each graph for visualization. **B.** Effects of MA on network strength were similar regardless of orientation session performance on the gradCPT. Dots represent individual participants, horizontal lines denote condition means, and error bars represent standard error of the mean.

bioRxiv preprint doi: https://doi.org/10.1101/2025.05.20.655181; this version posted May 21, 2025. The copyright holder for this preprint (which was not certified by peer review) is the author/funder, who has granted bioRxiv a license to display the preprint in perpetuity. It is made available under aCC-BY 4.0 International license.

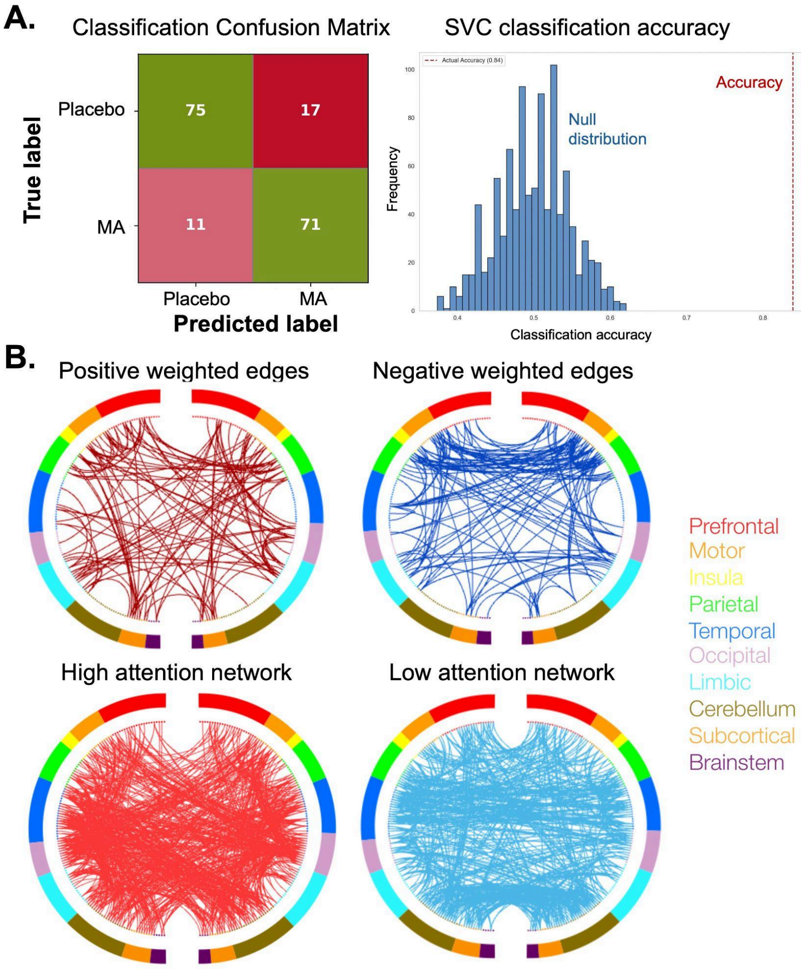

Figure S4. A. The support vector classifier distinguishes subjects in MA versus placebo conditions with an accuracy of 83.91%. **B.** The first row of circle plots shows the top 2.5% most positive and most negative weighted functional connectivity edges of the support vector classifier (SVC) in distinguishing between MA and placebo conditions. Networks were defined using functional connectivity patterns during the resting-state scan; similar results were observed for the Monetary Incentive Delay (MID) task and Doors task (Figure 5.1, 5.2). The second row of circle plots shows the high-attention and low-attention networks, defined to predict performance on an attention task in an independent group of participants (Rosenberg et al., 2016b). Nodes are grouped by macroscale regions, including the prefrontal cortex, motor cortex, insula, parietal cortex, temporal cortex, occipital cortex, limbic lobe (including the cingulate cortex, amygdala, and hippocampus), cerebellum, subcortex (thalamus and striatum), and brainstem. The right half of the circle represents the right hemisphere of the brain.

bioRxiv preprint doi: https://doi.org/10.1101/2025.05.20.655181; this version posted May 21, 2025. The copyright holder for this preprint (which was not certified by peer review) is the author/funder, who has granted bioRxiv a license to display the preprint in perpetuity. It is made available under aCC-BY 4.0 International license.

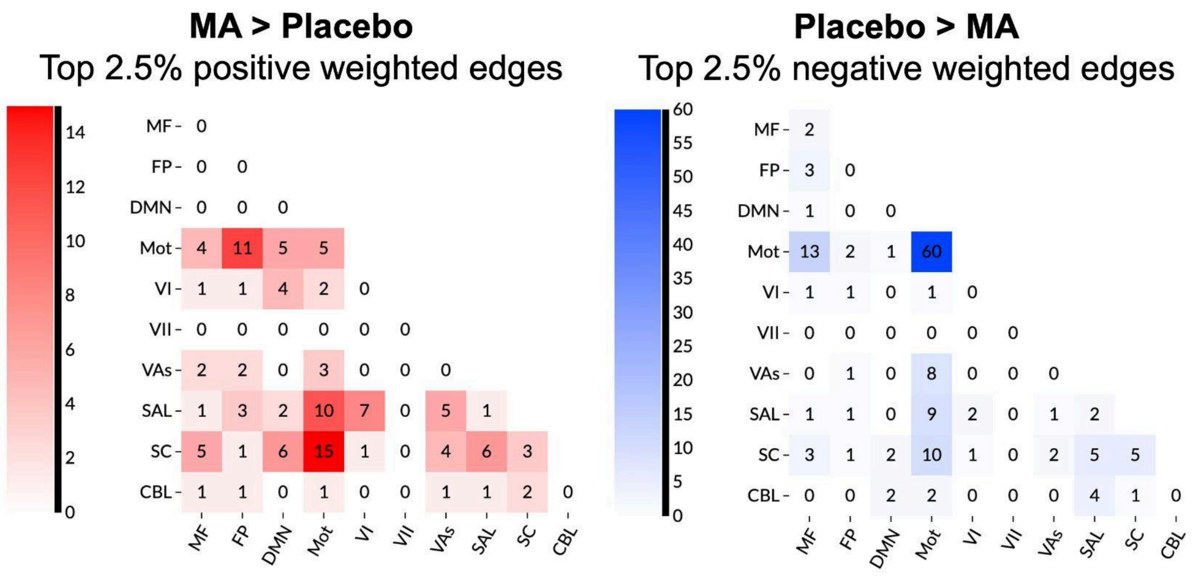

Figure S5. Top 2.5% positive and negative weighted edges in the MA network. Connections between the motor cortex and cerebellum, limbic regions, basal ganglia, and fronto-parietal networks were particularly influential for the classification.
